# An Impedance Sensor for Distinguishing Multi-Contaminants in Hydraulic Oil of Offshore Machinery

**DOI:** 10.3390/mi12111407

**Published:** 2021-11-17

**Authors:** Haotian Shi, Dian Huo, Hongpeng Zhang, Wei Li, Yuqing Sun, Guobin Li, Haiquan Chen

**Affiliations:** Marine Engineering College, Dalian Maritime University, Dalian 116026, China; dmu6hao@163.com (H.S.); hd116026@dlmu.edu.cn (D.H.); dmuliwei@dlmu.edu.cn (W.L.); sunyq@dlmu.edu.cn (Y.S.); chenapec@163.com (H.C.)

**Keywords:** impedance sensor, multi-contaminants, inductance-resistance mode, capacitance mode, offshore hydraulic machinery

## Abstract

The cleanliness of hydraulic oil can reflect the service life of the oil and the wear state of hydraulic machinery. An impedance sensor is proposed to distinguish multi-contaminants in hydraulic oil. The impedance sensor has two detection modes: the inductance-resistance mode is used to detect metal debris, and the capacitance mode is used to distinguish water droplets and air bubbles. By adding a built-in silicon steel strip and an external silicon steel strip with high magnetic permeability, the distribution area, strength, and uniformity of the magnetic field are enhanced to improve the detection sensitivity under inductance and resistance parameters. In addition, the silicon steel strips are used as electrode plates to introduce capacitance parameter detection. The experimental results show that the resistance detection method based on coil successfully improves the detection ability for non-ferromagnetic metal debris. The impedance sensor for distinguishing multi-contaminants in hydraulic oil can provide technical support for fault diagnosis of offshore hydraulic machinery.

## 1. Introduction

Hydraulic technology is widely used in the ocean engineering field, such as offshore drilling platforms, offshore wind turbine, and ships. According to statistics [1,2], hydraulic oil pollution is one of the main reasons for the abnormal operation of hydraulic machinery. Currently, oil detection technology has become a common method for monitoring and fault diagnosis of marine machinery. However, the oil condition monitoring of offshore machinery is still based on off-line detection methods, such as analysis of physicochemical properties, ferrograph analyzer [3], and spectrometer [4]. These methods can quantitatively analyze the physical and chemical state of oil, and obtain the types and contents of contaminants. However, due to the oil sample need to be sent to the laboratory for detection, it has the disadvantages of a long measurement cycle, high cost and low time efficiency. Compared with the onshore equipment, the offshore equipment has the characteristics of high strength and harsh environment [5]. If the failure caused by abnormal wear is not eliminated in time, it may lead to disastrous consequences and endanger ocean environment. Therefore, the development of on-line and portable detection devices for offshore machinery is imminent.

The contaminants (metal debris, air, water) carried in hydraulic oil can reflect the service life of the oil and the wear state of the hydraulic components. Metal debris can reflect the position and degree of wear [6]. Water will deteriorate the oil emulsion and aggravate the corrosion [7]. Air will cause system action delay and cavitation [8]. The cleanliness of hydraulic oil is a key indicator for the health assessment of the hydraulic system. It is very important for fault diagnosis and safe operation of equipment to obtain accurately the information reflected by the hydraulic oil. Currently, there are several methods for on-line oil monitoring: optical detection [9], acoustic detection [10], and impedance detection [11]. All the above methods obtain the contamination information according to the direction, amplitude and number of the output signal pulses generated by the contaminants passing through the detection region. In addition, the pollution level can be determined according to international standards.

Based on the difference in detection principles (conductivity, permittivity, permeability), impedance methods are divided into resistance detection [12], capacitance detection [13] and inductance detection [14]. Among them, the inductance detection, as a typical non-destructive detection method, has stable performance and can distinguish metal debris, thereby attracting widespread attention. The research focus on inductive oil monitoring devices is how to improve the detection sensitivity. According to the current research situation, it can be divided into four methods: optimizing the sensor structure, enhancing the magnetic field strength, using the high-precision acquisition circuit and signal processing analysis. Fan et al. designed a debris sensor with multiple inductive coils, which established a toroidal magnetic field to make each inductive coil locate near a stronger magnetic field, thereby improving the detection accuracy [15]. Feng et al. applied the two-stage automatic asymmetry compensation circuit on the triple-coil debris sensor to suppress the residual voltage caused by the excitation coils and resist the environmental fluctuations for high resolution [16]. Hong et al. proposed a method based on multi-window correlation to detect debris with variable velocities [17]. This method can obtain a higher signal-to-noise ratio, which is conducive to improving the detection ability and adaptability of the sensor. However, inductive sensors cannot detect water and air in oil, but the content of water and air is also very important to determine the oil replacement time. Inductance debris sensors can be combined with other technologies to expand the detection range and obtain more oil state information. Du et al. applied an ultrasonic sensor and an inductive sensor to detect non-metallic debris, ferrous metallic debris and non-ferrous metallic debris [18]. Zhang et al. designed oil sensors integrating inductance and capacitance sensing technology to detect metal debris and non-metallic contaminants, using parallel plate capacitor, cylindrical capacitor and fringe effect of capacitor to measure capacitance parameter [19,20,21].

The impedance sensor proposed in this paper has two detection modes: the inductance-resistance mode is used to detect metal debris, and the capacitance mode is used to distinguish water droplets and air bubbles. Many methods such as optimizing sensing unit structure, enhancing magnetic field strength and adopting coil-based resistance parameter are integrated to improve the detection ability of sensor. In addition, silicon steel strips are used as electrode plates to establish capacitance a sensing region with a high-quality electric field. The detection floor level experiments show that the impedance sensor can distinguish >28 μm iron debris, >70 μm copper debris, >160 μm air bubbles and >95 μm water droplets. The impedance sensor can be used as the core sensing element of the portable device for onsite monitoring, thereby obtaining the health condition of offshore hydraulic machinery.

## 2. Sensor Structure Design

**Figure 1:**
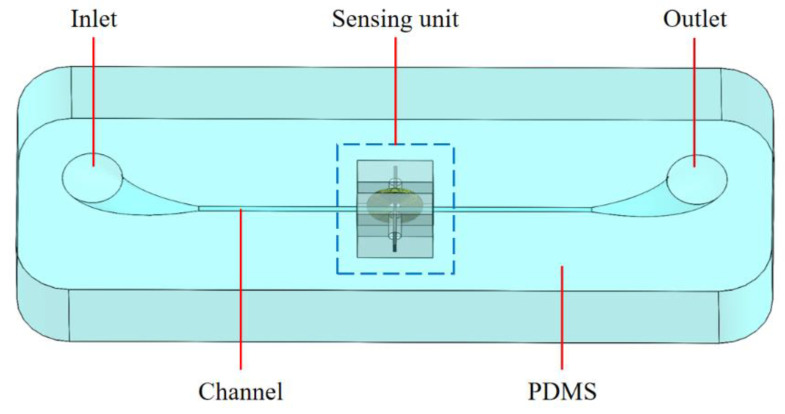
Overall structure diagram of impedance sensor.

**Figure 2:**
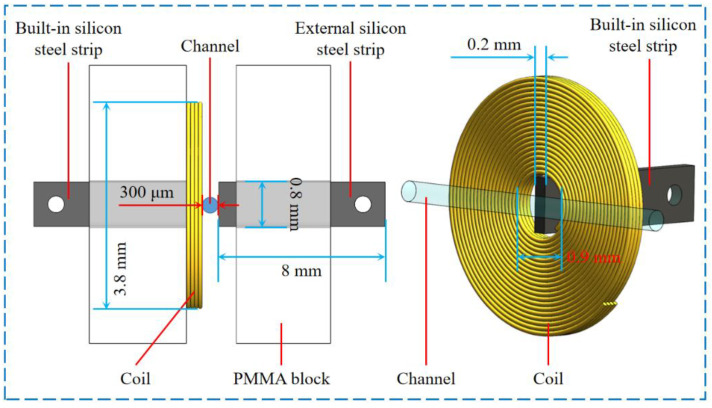
Structure of sensing unit.

The overall structure of the impedance sensor is shown in Figure 1, which includes the inlet, outlet, channel, polydimethylsiloxane (PDMS) substrate and sensing unit. The inlet and outlet adopt a tapered elbow structure to facilitate the injection of oil samples. The channel passes through the sensing unit, connecting the inlet and outlet. The transparent PDMS substrate can be used to observe contaminants passing through the sensing unit. The sensing unit is composed of coil, a built-in silicon steel strip and an external silicon steel strip, and two polymethyl methacrylate blocks for fixing the sensing elements. The coil with four layers and 80 turns is made of 70 μm diameter enameled wire. The end of the silicon steel strips is drilled with a hole for connecting leads. As shown in Figure 2, the built-in silicon steel strip is embedded in the coil’s inner hole, and the external silicon steel strip is arranged on the other side of the coil. The channel is close to the coil and the silicon steel strips, and the distance between the channel and the sensing element surface is 0 under ideal conditions. In the inductance-resistance mode, the terminals of the coil are connected with the excitation source to detect the metal debris. In the capacitance mode, the terminals of the silicon steel strips are connected with the excitation source to detect the air bubbles and water droplets. The distance between the two silicon steel electrodes is 300 μm.

## 3. Detection Principle

### 3.1. Inductance-Resistance Sensing Principle

The coil excited by alternating currents will produce an alternating magnetic field around it. When the metal debris passes through the magnetic field distribution region, the eddy current effect and magnetization effect will be stimulated inside the metal debris, which will break the original balance of the magnetic field, skin effect and proximity effect, resulting in the impedance change of the coil. According to the previous theoretical analysis [22], the impedance change caused by a single spherical metal debris is:

(1) $$Z_{d}=\frac{\oint_{\mathrm{coil}}-j\omega A_{d}\left(r\right)\cdot dl}{I}$$

Here, $j^{2}=-1$, ω is the angular frequency of excitation alternating current, dl is a vector about the differential element of the wire in the direction of the conventional current, I is the current in the coil. $A_{d}\left(r\right)$ is the magnetic vector potential distribution induced by the metal debris, which is given by:

(2) $$A_{d}\left(r\right)=\frac{v\chi_{d}}{4\pi }\left[B_{C}\left(r_{d}\right)+B_{B}\left(r_{d}\right)+B_{E}\left(r_{d}\right)\right]\times \frac{r-r_{d}}{{\left|r-r_{d}\right|}^{3}}$$

where v is the volume of metal debris, $\chi_{d}$ is magnetic susceptibility in an alternating magnetic field, $B_{C}\left(r_{d}\right)$ is the magnetizing field generated by the coil in the debris location, $B_{B}\left(r_{d}\right)$ is the magnetizing field generated by the built-in silicon steel strip in the debris location, $B_{E}\left(r_{d}\right)$ is the magnetizing field generated by the external silicon steel strip in the debris location, r is the arbitrary position vector of the coil, $r_{d}$ is the position vector of the debris center.

The resistance change of the sensing unit is:

(3) ΔR=Re(Zd)

The inductance change of the sensing unit is:

(4) ΔL=Im(Zdω)

The stronger magnetization effect of ferromagnetic metal debris will enhance the inductance of the sensing unit, the stronger eddy current effect of non-ferromagnetic metal debris will reduce the inductance of the sensing unit and the metal debris will change the current distribution of the sensing conductor and increase the AC resistance.

**Figure 3:**
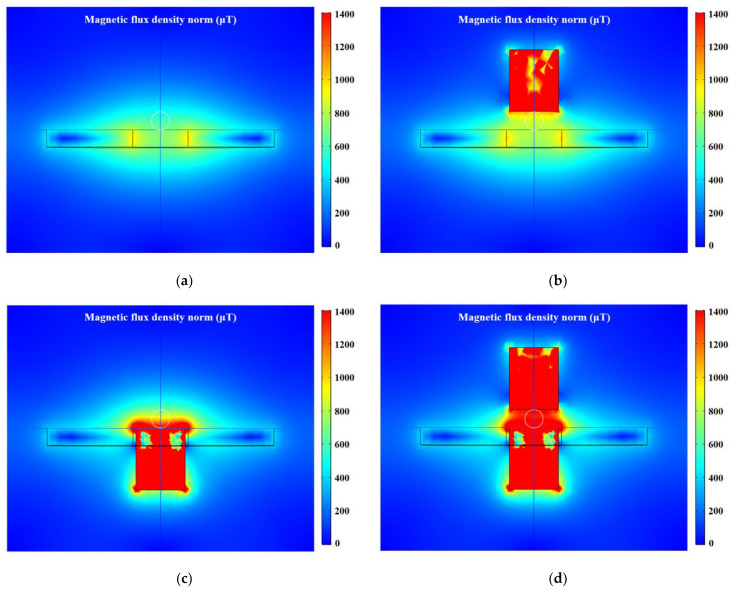
Distribution characteristics of magnetic field in detection region. (**a**) Magnetic field around coil. (**b**) Magnetic field around sensing unit with external silicon steel strip. (**c**) Magnetic field around sensing unit with built-in silicon steel strip. (**d**) Magnetic field around sensing unit with built-in and external silicon steel strips.

The distribution characteristics of the magnetic field in the detection region is simulated by COMSOL software, and the excitation applied to the coil is 2.0 V, 2.0 MHz. The distribution of the magnetic field generated by the coil is not uniform, and the magnetic field close to the inner hole edge is stronger, as shown in Figure 3a. Therefore, the impedance changes caused by the same metal debris in different positions are different. This phenomenon is called the particle position effect, which will reduce the detection accuracy of the sensor. High magnetic permeability materials can be added to enhance the magnetic field strength and uniformity in the detection region, thereby improving the stability of the sensor. The external silicon steel strip has a weak enhancement effect on the magnetic field, this is because the magnetizing field at the location of the external silicon steel strip is weak, as shown in Figure 3b. The built-in silicon steel strip is sufficiently magnetized, which greatly improves the magnetic field strength, but cannot completely cover the detection channel, as shown in Figure 3c. When both the built-in silicon steel strip and the external silicon steel strip exist, the magnetic field produced by the magnetization of the built-in silicon steel strip will further magnetize the external silicon steel strip. In this instance, the magnetic field strength at the channel location is increased from 600 μT to 1400 μT, and the distribution is more uniform, as shown in Figure 3d. The eddy current effect and magnetization effect inside the metal debris will increase with the magnetic field strength, so the impedance of the sensing unit will change more significantly after the silicon steel strips are added.

### 3.2. Capacitance Sensing Principle

The water droplets and air bubbles passing through the capacitance sensing region will cause the change of the mixture between the plates. According to the previous theoretical analysis [23], the capacitance change caused by contaminants is:

(5) ΔC=1ω⋅{Im(ZCm)[Im(ZCm)]2+[Re(ZCm)]2−Im(ZCo)[Im(ZCo)]2+[Re(ZCo)]2}

Here, $Z_{Cm}$ is the impedance when there are contaminants in the detection region, $Z_{Co}$ is the impedance when oil fills the detection region. The impedance of the parallel capacitor is:

(6) $$Z_{C}=\frac{d}{j\omega \tilde{\varepsilon }S}$$

where d is the distance between two electrode plates, S is the cross-sectional area of the electrode plate, $\tilde{\varepsilon }$ is the complex permittivity.

The equivalent complex permittivity of the mixture (oil and contaminants) ${\tilde{\varepsilon }}_{m}$ is

(7) $${\tilde{\varepsilon }}_{m}={\tilde{\varepsilon }}_{o}\frac{\left(1+2\varphi \right){\tilde{\varepsilon }}_{c}+\left(2-2\varphi \right){\tilde{\varepsilon }}_{o}}{\left(1-\varphi \right){\tilde{\varepsilon }}_{c}+\left(2+\varphi \right){\tilde{\varepsilon }}_{o}}$$

where ${\tilde{\varepsilon }}_{o}$ is the complex permittivity of hydraulic oil, ${\tilde{\varepsilon }}_{c}$ is complex permittivity of contaminant, φ is the ratio of the contaminant volume to the detection region volume.

**Figure 4:**
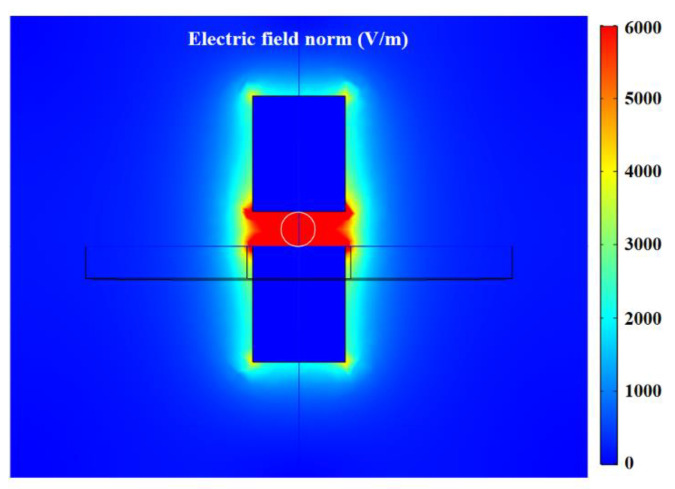
Distribution characteristic of electric field in detection region.

The complex permittivity of water is bigger than that of oil; water droplets will increase the capacitance value. The complex permittivity of air is smaller than that of oil; air bubbles will decrease the capacitance value. The distribution characteristics of the electric field in the detection region is simulated by COMSOL software (version COMSOL Multiphysics 5.4, COMSOL, Inc., Burlington, MA, USA), and the excitation applied to the silicon steel electrode plates is 2.0 V. As shown in Figure 4, the electric field distribution in the capacitance sensing region is uniform, completely covering the detection channel, and the electric field strength is 6000 V/m.

## 4. Experiment and Discussion

### 4.1. Sensor Performance Test System

**Figure 5:**
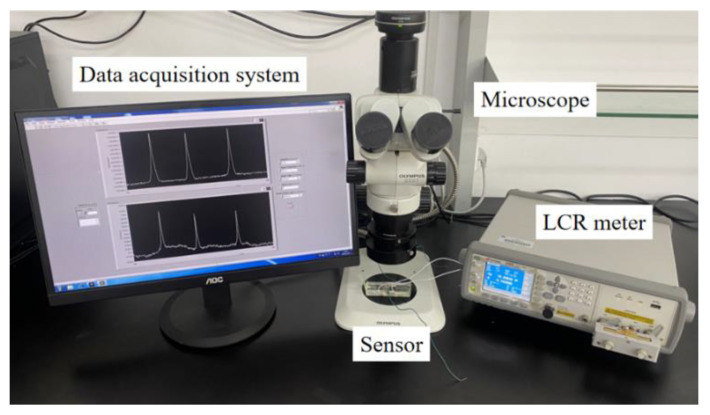
Photograph of test system.

The sensor performance test system was built, as shown in Figure 5. The LCR meter (Agilent E4980 A, Agilent Technologies Inc., Bayan Lepas, Malaysia) acts as an excitation source to apply excitation to the sensor, and measures the impedance change of the sensor. The impedance sensor can detect oil contaminants. The microscope is used to observe and measure the contaminants passing through the sensing region. The LabVIEW data acquisition system can read and store data in real time, and complete signal analysis. To obtain the impedance change caused by contaminants, the signal baseline is zeroed by an interpolation fit method for all signal diagrams.

### 4.2. Sensor Performance Test System

**Figure 6:**
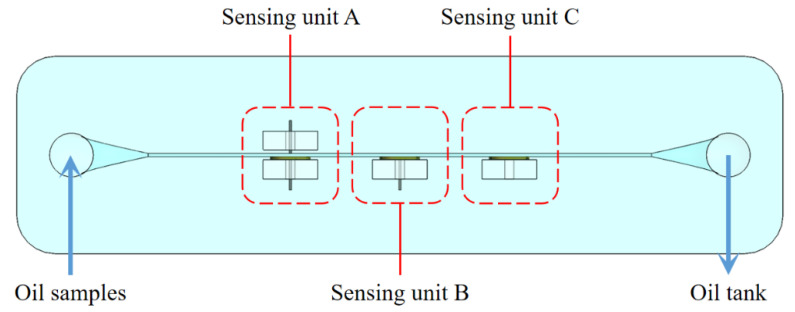
The sensor used for comparison experiments.

In the theoretical part, the function of the built-in silicon steel strip and the external silicon steel strip is studied through simulation analysis. To verify the simulation results, a sensor with three sensing units was made for comparison experiments, as shown in Figure 6. The built-in silicon steel strip and external silicon steel strip were added in sensing unit A, the built-in silicon steel strip was added in sensing unit B and there is no silicon steel strip in the sensing unit C. The 2 mg iron debris and 2 mg copper debris were uniformly mixed with 100 mL hydraulic oil to prepare the oil sample with metal debris. The oil sample with metal debris was driven by the syringe pump to enter the channel from the inlet, then flowed into the oil tank from the outlet. In addition, the same metal debris can pass through the sensing unit many times by changing the direction of the syringe pump. The sensor was switched to the inductance-resistance mode, and the excitation parameters were set to 2.0 V, 2.0 MHz.

**Figure 7:**
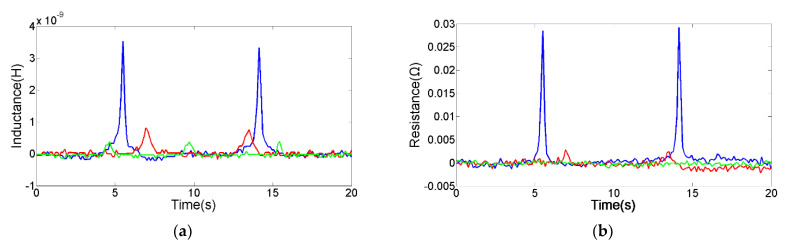
The detection results of 70 μm spherical iron debris, the green line is the signal from sensing unit C, the red line is the signal from sensing unit B, the blue line is the signal from sensing unit A. (**a**) Inductance signal diagram, (**b**) resistance signal diagram.

**Figure 8:**
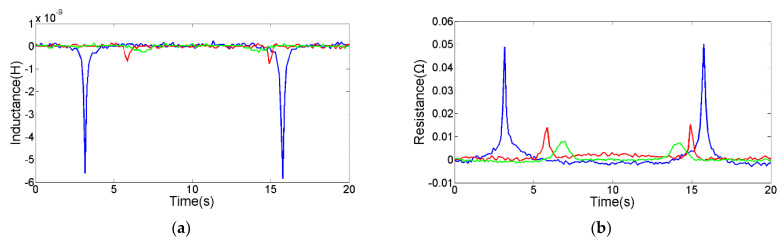
The detection results of 155 μm spherical copper debris, the green line is the signal from sensing unit C, the red line is the signal from sensing unit B, the blue line is the signal from sensing unit A. (**a**) Inductance signal diagram, (**b**) resistance signal diagram.

The 70 μm spherical iron debris and the 155 μm spherical copper debris were selected through a microscope for comparison experiments, as shown in Figure 7 and Figure 8. In addition, the pulse change values from different sensing units are shown in Table 1. The comparison experiment results show that the silicon steel strips can improve the detection sensitivity of the sensor. Especially when both the built-in silicon steel strip and the external silicon steel strip are added, the magnetic field is stronger and the distribution area is bigger, which is conducive to the larger electromagnetic disturbance generated by metal debris. In addition, the magnetic field is more uniform, avoiding the double-peak pulse generated by a single metal debris. Thereby, the detection capability of the inductance-resistance parameter is significantly enhanced. In addition, the silicon steel strips promote the rearrangement of skin effect and proximity effect, which further enhances the AC resistance change caused by metal debris.

### 4.3. Capacitance Experiments

In the capacitance mode, two silicon steel strips form a parallel plate capacitor for distinguishing water droplets and air bubbles in hydraulic oil. The 0.1 mL distilled water and 20 mL hydraulic oil were mixed in a sealed test tube, and the mixture was vibrated using an oscillator to prepare the oil sample with water droplets. The 0.1 mL air and 19.9 mL hydraulic oil were sealed in a 20 mL test tube, then the tube was placed in an ultrasonic oscillator to prepare the oil sample with air bubbles. The sensor was switched to the capacitance mode, and the excitation parameters were set to 2.0 V, 0.9 MHz.

**Figure 9:**
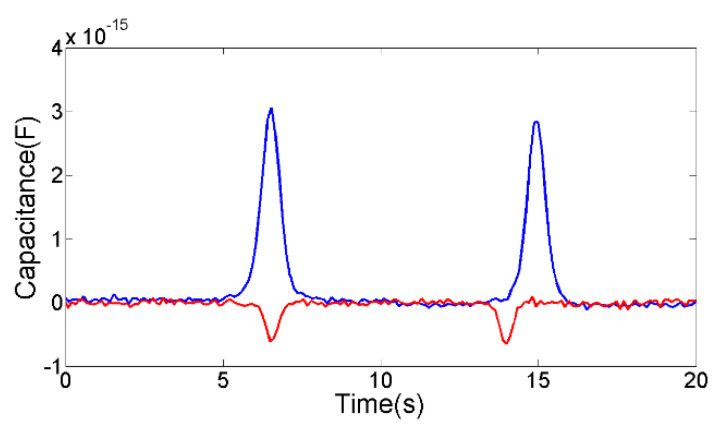
The capacitance detection results, the red line is the signal of 253 μm air bubble, the blue line is the signal of 245 μm water droplet.

The 253 μm air bubble and the 245 μm water droplet were selected through a microscope for experiments, as shown in Figure 9. The average capacitance pulse amplitude of 253 μm air bubble is −6.4 × 10^−16^ F, the average capacitance pulse amplitude of 245 μm water droplet is 2.96 × 10^−15^ F. According to the positive and negative of the capacitance change, the water droplets and air bubbles can be judged.

### 4.4. Research on Detection Floor Level of Sensor

**Figure 10:**
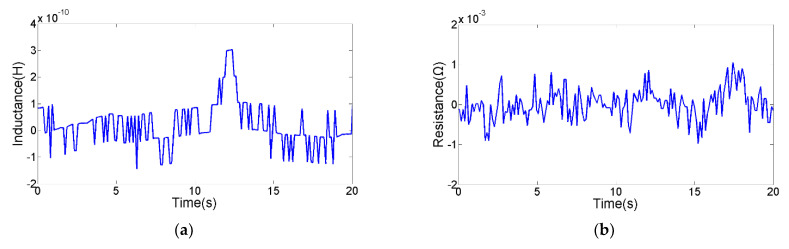

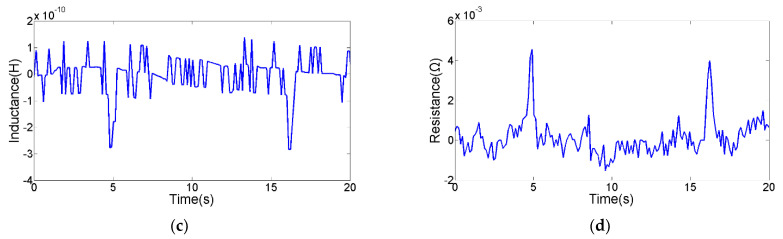
The detection floor level of inductance parameter. (**a**) The inductance signal of 28 μm iron debris, (**b**) the resistance signal of 28 μm iron debris, (**c**) the inductance signal of 85 μm copper debris, (**d**) the resistance signal of 85 μm copper debris.

**Figure 11:**
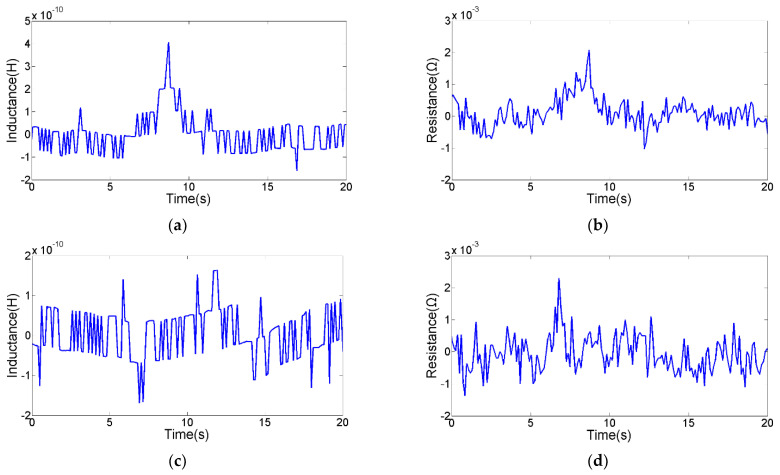
The detection floor level of resistance parameter. (**a**) The inductance signal of 40 μm iron debris, (**b**) the resistance signal of 40 μm iron debris, (**c**) the inductance signal of 70 μm copper debris, (**d**) the resistance signal of 70 μm copper debris.

**Figure 12:**
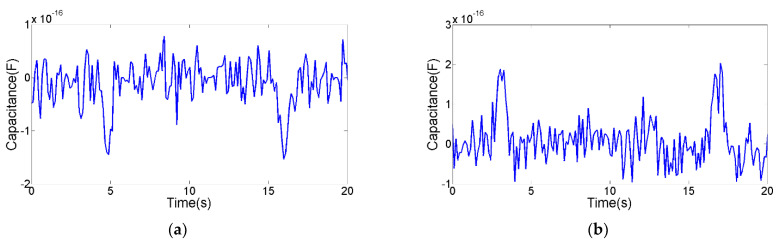
The detection floor level of capacitance parameter. (**a**) The capacitance signal of 160 μm air bubble, (**b**) the capacitance signal of 95 μm water droplet.

**Figure 13:**
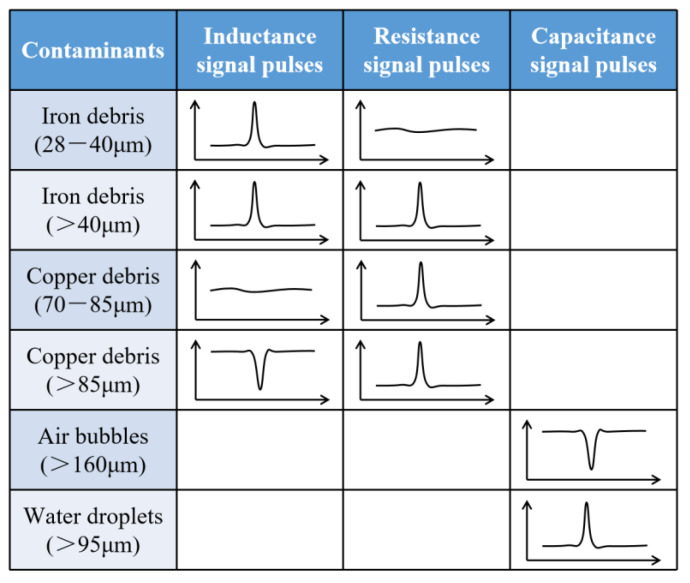
Signal pulse characteristics of contaminants with different sizes.

The detection floor level reflects the detection ability of the sensor. The detection floor levels of the inductance parameter, resistance parameter and capacitance parameter on oil contaminants were explored. The smallest contaminants that can be detected by inductance parameter are 28 μm iron debris and 85 μm copper debris, as shown in Figure 10. The smallest contaminants that can be detected by resistance parameter are 40 μm iron debris and 70 μm copper debris, as shown in Figure 11. The smallest contaminants that can be detected by capacitance parameter are 160 μm air bubble and 95 μm water droplet, as shown in Figure 12. The inductance pulse amplitude of 28 μm iron debris is 2.9 × 10^−10^ H, the inductance pulse amplitude of 85 μm copper debris is −2.8 × 10^−10^ H. The resistance pulse amplitude of 40 μm iron debris is 2.3 × 10^−3^ Ω, the resistance pulse amplitude of 70 μm copper debris is 2.4 × 10^−3^ Ω. The capacitance pulse amplitude of 160 μm air bubble is −1.4 × 10^−16^ F, the capacitance pulse amplitude of 95 μm water droplet is 1.9 × 10^−16^ F. The signal pulse characteristics of contaminants with different sizes can be obtained, as shown in Figure 13. The results show that the inductance parameter has higher detection sensitivity for iron debris, and resistance parameter has higher detection sensitivity for copper debris. In addition, the resistance detection method based on the coil successfully reduced the detection floor level of copper debris from 85 μm to 70 μm, which improved the detection ability for non-ferromagnetic metal debris. Based on the detection results from the two detection modes, >28 μm iron debris, >70 μm copper debris, >160 μm air bubbles and >95 μm water droplets can be distinguished.

This impedance microsensor has the advantages of high sensitivity and particle counting. In practical application, a portable oil monitoring device is integrated with the oil driving unit, oil processing unit, sensing unit (impedance microsensors), signal acquisition circuit, data analysis and display unit. The oil processing unit is used to filter the large-size impurities in oil samples to prevent the detection channel from being blocked. A high-precision measurement circuit needs to be designed to improve the anti-interference ability. A data analysis unit can obtain the contaminant information about type, size and amount.

## 5. Conclusions

An impedance sensor is proposed to comprehensively distinguish multi-contaminants in hydraulic oil. The impedance sensor has inductance-resistance and capacitance detection modes, which can distinguish metal debris, water droplets and air bubbles. By adding a built-in silicon steel strip and an external silicon steel strip with high magnetic permeability, the distribution area, strength and uniformity of the magnetic field are enhanced to stimulate stronger magnetization and eddy current effects inside the metal debris. Comparison experiments show that the addition of silicon steel strips significantly enhances the detection sensitivity under inductance and resistance parameters. In addition, in the capacitance mode, the built-in silicon steel strip and the external silicon steel strip as the electrode plates are used to distinguish water droplets and air bubbles. The detection floor level experiments show that the inductance parameter has a higher detection sensitivity for iron debris, and the resistance parameter has a higher detection sensitivity for copper debris. The resistance detection method based on the coil successfully reduced the detection floor level of copper debris from 85 μm to 70 μm, which improved the detection ability for non-ferromagnetic metal debris. Based on the detection results from the two detection modes, >28 μm iron debris, >70 μm copper debris, >160 μm air bubbles and >95 μm water droplets can be distinguished. The impedance sensor based on micromachining technology has the advantages of a simple structure, low cost and easy integration, which can be used as the core sensing element of the portable device for onsite monitoring. This technology can provide support for fault diagnosis of offshore hydraulic machinery.

## Figures and Tables

**Table 1:** The pulse change values from different sensing units.

Debris	Signal Type	Sensing Unit A	Sensing Unit B	Sensing Unit C
70 μm spherical iron debris	Average inductance pulse amplitude	3.43 × 10^−9^ H	7.8 × 10^−10^ H	4.1 × 10^−10^ H
	Average resistance pulse amplitude	2.86 × 10^−2^ Ω	3.0 × 10^−3^ Ω	Unrecognizable
155 μm spherical copper debris	Average inductance pulse amplitude	−5.84 × 10^−9^ H	−7.6 × 10^−10^ H	−3.0 × 10^−10^ H
	Average resistance pulse amplitude	5.03 × 10^−2^ Ω	1.41 × 10^−2^ Ω	7.8 × 10^−3^ Ω
